# Rubber Litigation

**Published:** 1874-06

**Authors:** 


### THE RUBBER LITIGATION.

There is perhaps no subject that so much interests and excites the greater part of the dentists of this country as the contest, that has been for years, and is still going on, relative to the right to use vulcanized rubber for dental purposes.

A similar contest in reference to the use of gold for the same purpose, would hardly have elicited as much interest.

The case of the Goodyear Dental Vulcanite Co., *et al.*, *vs.* Daniel H. Smith has attracted the attention of the entire profession for some time past.

This case was in preparation for some time and it was supposed, that in it, a strong defense would be made, and we believe that everything was done, that in this case, it was possible to do. But after all it failed, to the great disappointment of the profession.

The case was argued about three months ago before Hon. Geo. F. Shipley and the decision was rendered a few days ago, in favor of the Plaintiff. Whether this case will go to the Supreme Court we are not advised.

Another case by the same complainants *vs* Dr. Willis of Michigan, is to be argued in Detroit, about the 4th of June, for which thorough preparation has been made, and strong hope of success is entertained in it, for the profession. The Dentists should bear in mind that these cases, have not been carried on without great expense and they should see to it, that the money for this purpose is amply supplied.

